# Efficacy and safety of traditional Chinese medicine yangxin anshen therapy for insomnia: A protocol of systematic review and meta-analysis

**DOI:** 10.1097/MD.0000000000016945

**Published:** 2019-09-13

**Authors:** Feizhou Li, Xuanxuan Wang, Ziyu Song, Ling Liu, Tong Zhang, Yanhua Chen, Ping Wang

**Affiliations:** a Clinical College of Traditional Chinese Medicine; b Institute of Gerontology, Hubei University of Chinese Medicine; c Hubei Cancer Hospital; d The First Clinical College, Hubei University of Chinese Medicine; e Encephalopathy Department, Hubei Provincial Hospital of Traditional Chinese Medicine, Wuhan City, Hubei Province, China.

**Keywords:** insomnia, meta-analysis, protocol, systematic review, traditional Chinese medicine, yangxin anshen therapy

## Abstract

**Background:**

Traditional Chinese medicine (TCM) has gradually drawn the attention of clinicians as an alternative choice for insomniacs and TCM yangxin anshen therapy (TYAT), as a crucial therapy of treating insomniacs, is based on the theory of syndrome differentiation. However, owing to the lack of evidence-based medical evidence, the authors intend to carry out this study to evaluate TYAT's effectiveness and safety.

**Methods:**

Seven electronic databases will be searched from inception to July 2019. Two authors will independently identify randomized controlled trials, fetch data and assess the risk of bias with tools provided by Cochrane. A comprehensive meta-analysis will be conducted with the Cochrane Collaboration software (Review Manager 5.3) for eligible and appropriate studies. Further, the evidence will be assessed with the grading of recommendations assessment, development, and evaluation approach.

**Results:**

This article will be dedicated to assessing TYAT's efficacy and safety for insomniacs.

**Conclusion:**

This systematic review and meta-analysis may provide persuasive evidence for the clinical application of TYAT in treating insomnia.

**Trial registration number:**

PROSPERO CRD 42019135115.

## 1 Introduction

Insomnia refers to a subjective experience that feels dissatisfied with sleep duration or quality and affects daytime social functioning despite appropriate sleep opportunities and sleep environments. In industrialized countries, about 6% of adults are afflicted with chronic insomnia.^[1]^ A cross-border European study among the elderly in 16 European countries found that the prevalence of insomnia ranges from 16.6% in Denmark to 31.2% in Poland.^[2]^ It is estimated that the proportion of the general population suffer from insomnia in China is about 15%, which is on the low side compared with the western countries but is close to reports from Asian countries.^[3]^ At the same time, the threat posed by insomnia to human health has gradually gained attention. Studies have found that chronic insomnia increases the risk of cardiovascular disease, especially the reduction of objective sleep duration was found to be a risk factor for hypertension.^[4,5]^ According to a prospective study,^[6]^ both difficulty initiating sleep and nonrestorative sleep are associated with a modestly higher risk of mortality. Besides, insomnia also places a substantial socioeconomic burden.^[7,8]^ Currently, clinical medications for insomniacs in China mainly include benzodiazepine drugs, nonbenzodiazepine drugs, melatonin receptor agonists, orexin receptor antagonist, along with the antidepressant drugs with hypnotic effects.^[9]^ However, these drugs have limitations of one or more undesirable adverse effects, such as the dizziness, headache, forgetfulness, bitter mouth, fatigue, withdrawal reaction, lethargy, hangover, falls, and so forth.^[10]^

Looking back on the past over 2000 years of history, herbal medicines have been extensively used to treat insomniacs in China. According to the theory of traditional Chinese medicine (TCM), the heart (“Xin” in Chinese Pinyin is used in this paper to distinguish it from the anatomically described organ) is thought to be related to the pathogenesis of insomnia in line with its function of the “seat of consciousness” and the place where the spirit or mind (“Shen” in Chinese Pinyin) is located, which can guide the follow-up treatments.^[11]^ Based on this, “Xin” is considered to be closely related to the occurrence of insomnia and TCM yangxin anshen therapy (TYAT), which can be defined as the therapy of tranquilizing the mind by nourishing the heart, is one of the crucial therapeutic principles for insomnia in TCM.

Previous meta-analyses^[12,13]^ have suggested that Chinese herbal medicine is a potential alternative therapy for insomnia. However, due to the heterogeneity of different studies, the evidence cannot conclusively confirm the feasibility of the clinical application of herbs in insomniacs. Given that the above meta-analysis did not take the differences of TCM therapeutic principles among studies into account, the authors plan to conduct this study to provide evidence-based medical evidence for the clinical application of TYAT for insomniacs.

## 2 Methods

### 2.1 Protocol register

This protocol of systematic review and meta-analysis has been drafted under the guidance of the preferred reporting items for systematic reviews and meta-analyses protocols statement^[14]^ and has already been registered on the PROSPERO platform (https://www.crd.york.ac.uk/PROSPERO/) with an assigned registration number CRD42019135115.

### 2.2 Ethics

Since this is a protocol with no patient recruitment and personal information collection, no further ethical approval is required.

### 2.3 Database search strategy

Computer retrieval and manual retrieval will be used to retrieve all the published literature independently by 2 authors. Databases searched include China National Knowledge Infrastructure, Chinese Scientific Journals Database, Wanfang Database, China Biological Medicine Database, PubMed, EMBASE Database and Cochrane Central Register of Controlled Trials. All randomized controlled trials (RCTs) on TYAT for insomnia will be collected from inception of each database to July 2019. The specific search strategy will be formulated with the specific database. Among them, the author lists the retrieval strategies of the PubMed database (see Table 1). It will also be supplemented by a manual search of relevant literature.

**Table 1:**
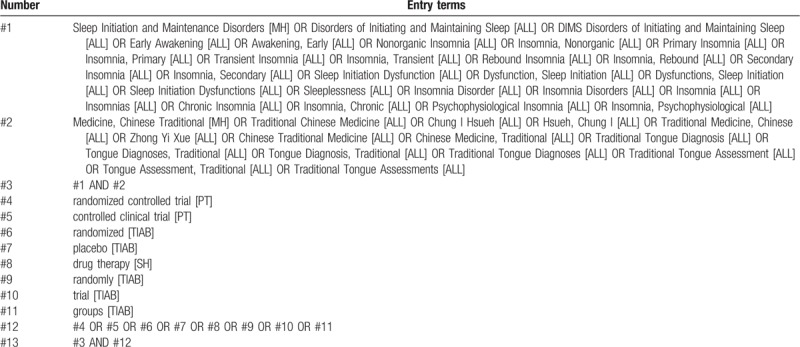
PubMed search strategy draft.

### 2.4 Eligibility criteria and elimination criteria

#### 2.4.1 Types of participants

The included patients should be explicitly diagnosed with insomnia, regardless of gender, ethnic background, or nationality. All subjects must meet the corresponding criteria: diagnostic and statistical manual of mental disorders,^[15]^ international classification of diseases,^[16]^ Chinese classification and diagnostic criteria of mental disorders,^[17]^ or Chinese guideline for the diagnosis and treatment of insomnia in adults.^[9]^ Studies involving other diseases will be ruled out, such as severe mental illness, drug laziness, and so forth.

#### 2.4.2 Types of interventions

Interventions assigned to the experimental groups must be an oral prescription that embodied the therapeutic principles of TYAT. The intervention in the control group could be benzodiazepines, nonbenzodiazepine hypnotics, placebo, or other basic treatment. The dose, dosage form, and treatment duration of the 2 groups will not be taken into considerations in this study. Studies involving acupuncture, moxibustion, massage, and other TCM prescription will be eliminated. Additionally, the authors are about to eliminate studies involving unfixed TCM prescriptions.

#### 2.4.3 Types of outcome measures

The primary outcome measures adopted in this study are polysomnography and the Pittsburgh sleep quality index (PSQI).^[18]^ The secondary outcome measures are clinical efficiency and adverse events. It is supposed to be noted that the criteria for clinical efficiency are divided into TCM curative efficacy^[19,20]^ and PSQI curative efficiency^[21]^ in our study.

#### 2.4.4 Type of study

RCTs that focused on TYAT for sleep disorders, whether or not blinding, will be included. Only Chinese and English literature will be taken into account. However, quasi-RCTs enrolled according to medical record number or birthday will not be set out in the present paper.

### 2.5 Study selection and data collection

**Figure 1:**
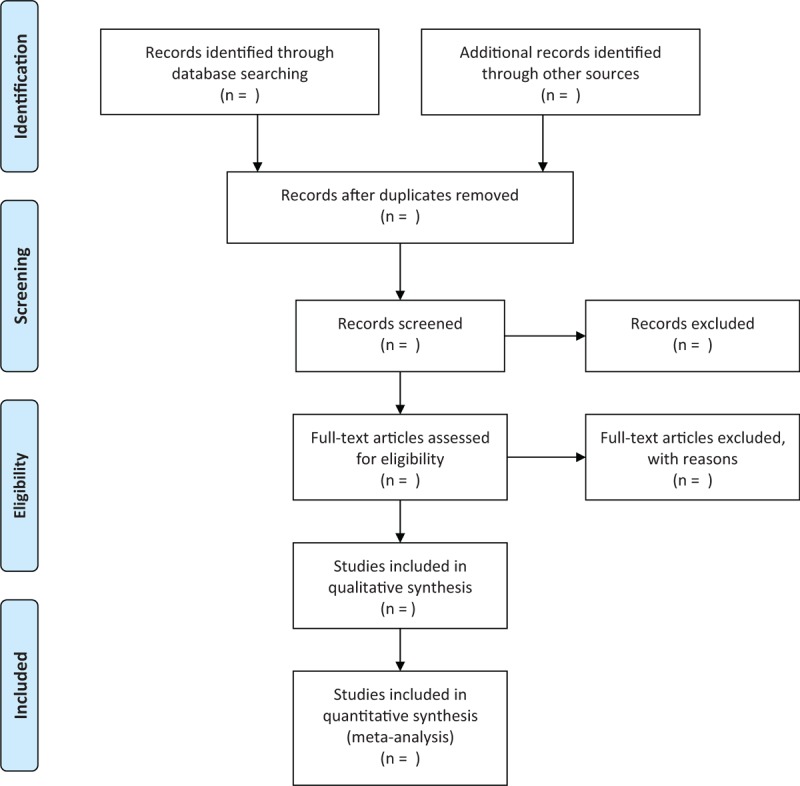
Flowchart of study screening.

Two investigators are in charge of completing the literature search according to the search strategy, respectively. Retrieved articles will be included in the form of titles and abstracts for further screening. The reasons for literature rejection will be recorded separately. In case of disagreement between the 2 investigators, they should settle through negotiation first. If necessary, the third investigator ought to be invited for determination. The flow chart is presented in Figure 1. Finally, 2 authors are expected to extract the data of included literature respectively to make a characteristic table, which includes the first author, year of publication, diagnostic criteria, sample size, interventions, the course of treatment, outcome measures, and follow-up time.

### 2.6 Literature quality assessment

The quality evaluation of the RCTs to be included will be completed using the tools recommended by the Cochrane Collaboration.^[22]^ The judgment of bias risk includes 7 aspects: random sequence generation, allocation concealment, blinding of participants and personnel, blinding of outcome assessment, incomplete outcome data, selective reporting, and other bias. The score of each article is distributed from 0 to 7. Two investigators will conduct the quality evaluation separately and invite a third reviewer to deal with any disagreement.

### 2.7 Statistical analysis methods

The Review Manager 5.3 software will be implemented for statistical analysis.^[23]^ Dichotomous data will be analyzed by using the risk ratio with 95% confidence interval (CI) and continuous variables will be analyzed by using the mean difference with 95% CI. For statistical heterogeneity, chi-square test and *I*^2^ index for measurements are used. If there is no statistical heterogeneity among the studies (*P* > .10, *I*^2^ < 50%), the fixed-effect model will be employed. If there is statistical heterogeneity among studies (*P* ≤ .10, *I*^2^ ≥ 50%), the sources of heterogeneity will be analyzed, and subgroup analysis may be undertaken. If there is statistical heterogeneity but no clinical heterogeneity among studies, the random effect model will be employed for analysis. If heterogeneity obviously exists between studies, only the descriptive analysis will be promoted. Sensitivity analysis will be carried out by changing the effect model and statistical methods. Only when the subgroup included >10 studies, publication bias can be measured.

### 2.8 Grading of recommendations assessment, development, and evaluation quality assessment

This assessment will be carried out through the Guideline Development Tool (https://gradepro.org/). On account of grading of recommendations assessment, development, and evaluation handbook,^[24]^ 2 independent authors will classify the quality of evidence into the following 4 levels: high quality, moderate quality, low quality, and very low quality.

## 3 Discussion

TCM syndrome (TS), the basic characteristics and a core concept, refers to a unique diagnostic method for classifying different individual pathological conditions under the guidance of TCM theory, which can be regarded as subtypes of modern diseases. Differentiation of TS, a basic feature that runs through the development history of TCM for more than 3000 years, is the basis for all TCM doctors to carry out clinical diagnosis and treatment.^[25]^ Differentiation of TS mainly based on clinical features would further contribute to classifying patients to improve the curative efficacy of TCM-associated therapeutic measures.^[26]^ In a nutshell, each kind of TS corresponds to its own therapy and making therapeutic regime based on TS differentiation is the key to enhance clinical efficacy.

As mentioned earlier, previous several meta-analyses^[12,13]^ did not take the differences of TCM therapies among studies into account. Therefore, there is a necessity to comprehensively retrieve and rigorously carry on high-quality research. To some extent, our appropriate protocol designed ahead may ensure that future evaluations of specific therapy for insomniacs like TYAT are more likely to provide objective evidence of guiding value. For all we know, this study will be the first attempt to evaluate the evidence of TYAT in the treatment of insomniacs.

It has to be acknowledged that some restrictions may affect the drawn conclusion based on this protocol. Retrieved studies will only include those published in Chinese or English. If certain differences in the diagnose criteria, interventions, dosage, duration of medication, and so forth could be not properly addressed, excessive heterogeneity may result, which may affect the conclusion.

## Author contributions

**Conceptualization:** Feizhou Li, Ping Wang.

**Data curation:** Feizhou Li, Xuanxuan Wang.

**Formal analysis:** Feizhou Li, Tong Zhang.

**Methodology:** Ziyu Song, Yanhua Chen.

**Project administration:** Ping Wang.

**Supervision:** Ling Liu, Ping Wang.

**Validation:** Ling Liu, Ping Wang.

**Writing – original draft:** Feizhou Li, Xuanxuan Wang, Ziyu Song, Tong Zhang, Yanhua Chen.

Feizhou Li orcid: 0000-0002-9465-1001.
